# Systematic Reviews of Animal Studies; Missing Link in Translational Research?

**DOI:** 10.1371/journal.pone.0089981

**Published:** 2014-03-26

**Authors:** Judith van Luijk, Brenda Bakker, Maroeska M. Rovers, Merel Ritskes-Hoitinga, Rob B. M. de Vries, Marlies Leenaars

**Affiliations:** 1 SYRCLE - Central Animal Laboratory, Radboud University Medical Centre, Nijmegen, the Netherlands; 2 Departments for Health Evidence and Operating rooms, Radboud University Medical Centre, Nijmegen, the Netherlands; University of Münster, Germany

## Abstract

**Background:**

The methodological quality of animal studies is an important factor hampering the translation of results from animal studies to a clinical setting. Systematic reviews of animal studies may provide a suitable method to assess and thereby improve their methodological quality.

**Objectives:**

The aims of this study were: 1) to evaluate the risk of bias assessment in animal-based systematic reviews, and 2) to study the internal validity of the primary animal studies included in these systematic reviews.

**Data Sources:**

We systematically searched Pubmed and Embase for SRs of preclinical animal studies published between 2005 and 2012.

**Results:**

A total of 91 systematic reviews met our inclusion criteria. The risk of bias was assessed in 48 (52.7%) of these 91 systematic reviews. Thirty-three (36.3%) SRs provided sufficient information to evaluate the internal validity of the included studies. Of the evaluated primary studies, 24.6% was randomized, 14.6% reported blinding of the investigator/caretaker, 23.9% blinded the outcome assessment, and 23.1% reported drop-outs.

**Conclusions:**

To improve the translation of animal data to clinical practice, systematic reviews of animal studies are worthwhile, but the internal validity of primary animal studies needs to be improved. Furthermore, risk of bias should be assessed by systematic reviews of animal studies to provide insight into the reliability of the available evidence.

## Introduction

The majority of animal experiments is being carried out in the context of preclinical research, e.g. to test safety and efficacy of new treatments to improve healthcare. However, translating animal data to the human situation has been proven to be very challenging. Various factors influence this translation, such as biological differences between species, internal validity, differences in experimental design between animal studies and clinical trials, insufficient reporting, and publication bias [1]. Systematic reviews (SRs) of animal studies have the potential to reduce some of the challenges in the translation of animal data to clinical trials, for example by explicitly assessing the internal validity. SRs attempt to identify, appraise and synthesize all the empirical evidence that meets pre-specified eligibility criteria to answer a given research question. SRs of animal studies are still quite rare, but their number appears to be slightly increasing [2]–[4].However, little is known about the extent to which the available SRs include a risk of bias assessment, in which the internal validity of the included primary animal studies is evaluated. We therefore performed a systematic review of the risk of bias assessment in SRs of animal studies. Subsequently, we studied the internal validity of the individual studies included in these SRs.

## Materials and Methods

### Search Strategy

To find all SRs of animal studies published between 2005 and 2012, the following search strategy was carried out on 28 January 2013. To identify animal studies, the MEDLINE (PubMed platform) and EMBASE (OvidSP platform) databases were searched using the ‘Animal’ filter for PubMed by Hooijmans et al., [5] and the filter for EMBASE by De Vries et al., [6], [7] respectively. Since we were interested in SRs, we used the clinical query for SRs from PubMed, which we have adapted for Embase (see S1).

### Study Selection

For the purpose of this study, a review was classified as a SR when at least all of the following items were reported: 1) the term Systematic Review 2) database(s) searched and 3) search terms. Selection was performed by two independent observers and disagreements were resolved through discussion (JvL, BB, ML). Only SRs aiming to inform human healthcare by reviewing a medical drug intervention were included, such as vitamin-based supplementations or stem cells treatment. Medical devices, such as prosthetics and scaffolds, and other types of interventions such as oxygen or heat were excluded. We also excluded SRs that were not written in English or could not be retrieved in full text. When supplementary data were available online, these were obtained.

### Scoring Procedure of SRs

Data on both the characteristics and methods used to assess risk of bias in the SRs were extracted by at least two independent reviewers (JvL, BB, ML). In SRs where both animal and human studies were included in the SR, only the animal data were evaluated. Disagreements between reviewers were resolved through discussion and if necessary a third reviewer was consulted.

#### Assessment risk of bias items

The methodology of quality assessment differs between SRs of animal studies [8]. For the purpose of this study we focused on the internal validity of primary studies. Therefore, we defined quality assessment as a risk of bias assessment. To fit this definition, the assessment had to include at least one of the following internal validity items: 1) randomized study design (selection bias), 2) blinding of investigator/caretaker (performance bias), 3) blinding of outcome assessment (detection bias) and 4) mentioning of drop-outs (attrition bias).

#### SR characteristics

Additional information on the characteristics of the SRs was extracted: 1) the way in which the risk of bias was taken into account in the SR (e.g. conduct of subgroup analyses based on quality, exclusion of studies based on quality or a general comment/statement related to the study quality), 2) level of reporting detail on internal validity (e.g. score per item or a summary for quality per study) and 3) research area of the SR.

### Data Extraction Primary Studies

SRs that provided detailed information on the required internal validity items were used to evaluate the internal validity of the included individual studies. Per SR, data were extracted on: total number of included studies and number of studies per item (randomised study design, blinding investigator/caretaker, blinding outcome assessment and drop-outs).

## Results

### Literature Search and SR Selection

**Figure 1:**
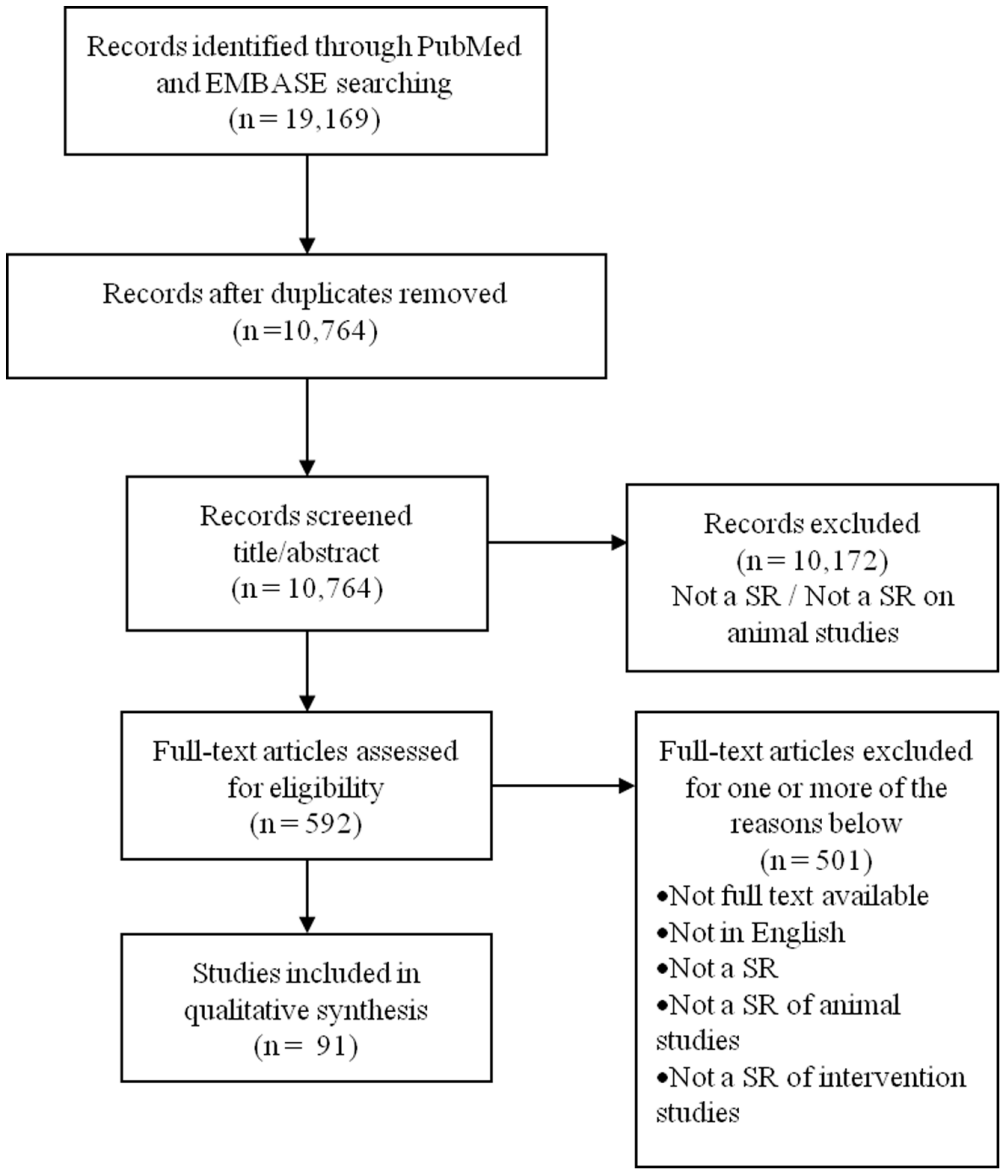
Flow diagram the systematic review literature search results. A total of 91 systematic reviews of intervention animal studies were included.

We identified 592 potentially eligible articles, of which 91 SRs met our inclusion criteria. Figure 1 shows the number of studies identified at each stage of the selection process. A complete list of the 91 references can be found elsewhere (see Table S1 in File S2).

### Characteristics of Included Systematic Reviews

**Figure 2:**
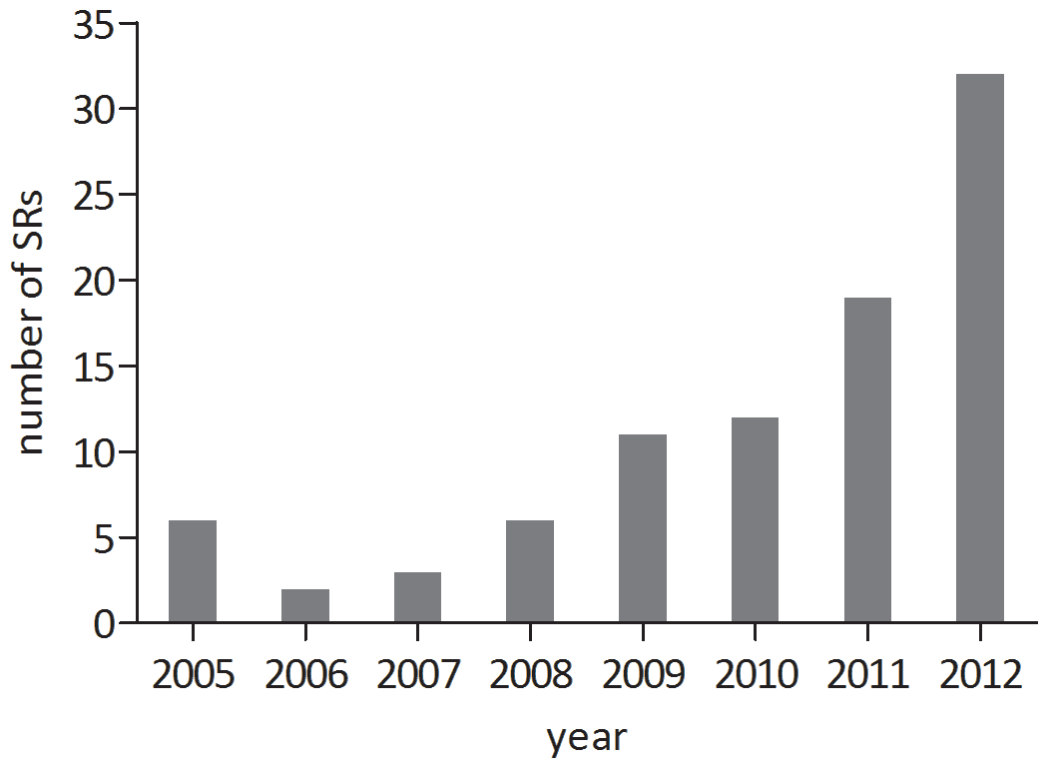
Number (n) of published SRs of intervention animal studies per publication year (2005–2012).

The number of published systematic reviews of animal studies increased over the last years from 6 in 2005 and 2, 6 and 12 in 2006, 2008 and 2010 to 32 in 2012, respectively (Figure 2).

The 91 SRs included in this review cover a range of research topics. Most reviews (n = 38; 41.8%) cover a neurological topic, of which 20 reviews (22.0%) pertained to stroke. The second largest group was on endocrinology (n = 11; 12.1%). Other topics included cardiovascular diseases, orthopaedics, infectious diseases, oncology, pharmacotoxicology, dentistry and gastroenterology. The complete list of topics and number of SRs per topic can also be found elsewhere (see Table S1 in File S2).

### Risk of Bias Assessment

#### Risk of bias assessment in systematic reviews

**Figure 3:**
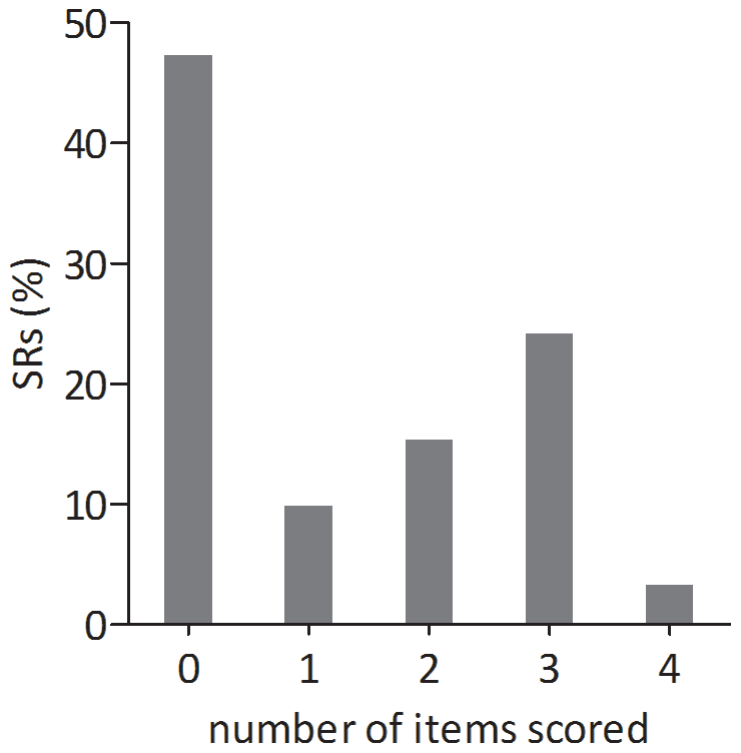
Percentages of SRs per number of internal validity item scored. Zero items by 47.3%, one item all randomisation, two items randomisation and one level of blinding, three items randomisation, blinding of caretaker/investigator and blinding of outcome assessment or randomisation, one level of blinding and drop-outs) and all four items by 3.3%.

Nearly half of the SRs (n = 43; 47.3%) did not assess any of the risk of bias items (figure 3). In 48 reviews (52.7%), one or more of our predefined risk of bias items were assessed. Thirty-three (36.3%) reviews also provided detailed information on the outcome of this assessment per individual study.

Figure 3 shows that of the 91 SRs only 3 (3.3%) assessed all 4 internal validity items in their quality assessment. Twenty-two SRs (24.2%) assessed 3 items, of which 17 SRs (18.7%) did not assess drop-outs; the other 5 (5.5%) did not score blinding of the caretaker. Fourteen SRs (15.4%) assessed two items namely randomisation and blinding (of these, 13 SRs assessed blinding of the outcome assessment, in one SR the type of blinding was unclear). Nine SRs (9.9%) assessed only one item, which in all cases was randomisation.

#### Risk of bias use in SRs

Of the 48 SRs that assessed risk of bias of included individual studies, 45 (93.8%) referred to the internal validity of the primary studies in the results, discussion or conclusion section. This means that three SRs did not discuss the outcome of the risk of bias assessment in any way. In most reviews, (n = 42; 87.5%) a general comment was made on the quality of the primary studies. In 25 SRs (52.1%), the primary study quality was used as a factor in the meta-analysis (e.g. subgroup analyses) and in three SRs the study quality was used as an exclusion criterion (see Table S1 in File S2).

### Internal Validity of Primary Studies

Thirty-three SRs that provided detailed information on the risk of bias assessment were used to evaluate the internal validity of the included primary studies. These 33 SRs included a total of 2280 primary studies (median 18, range: 2 – 1152 primary studies). Most of these studies were on the subject of stroke or other neurological topics (see Table S1 and S2 in File S2).

**Figure 4:**
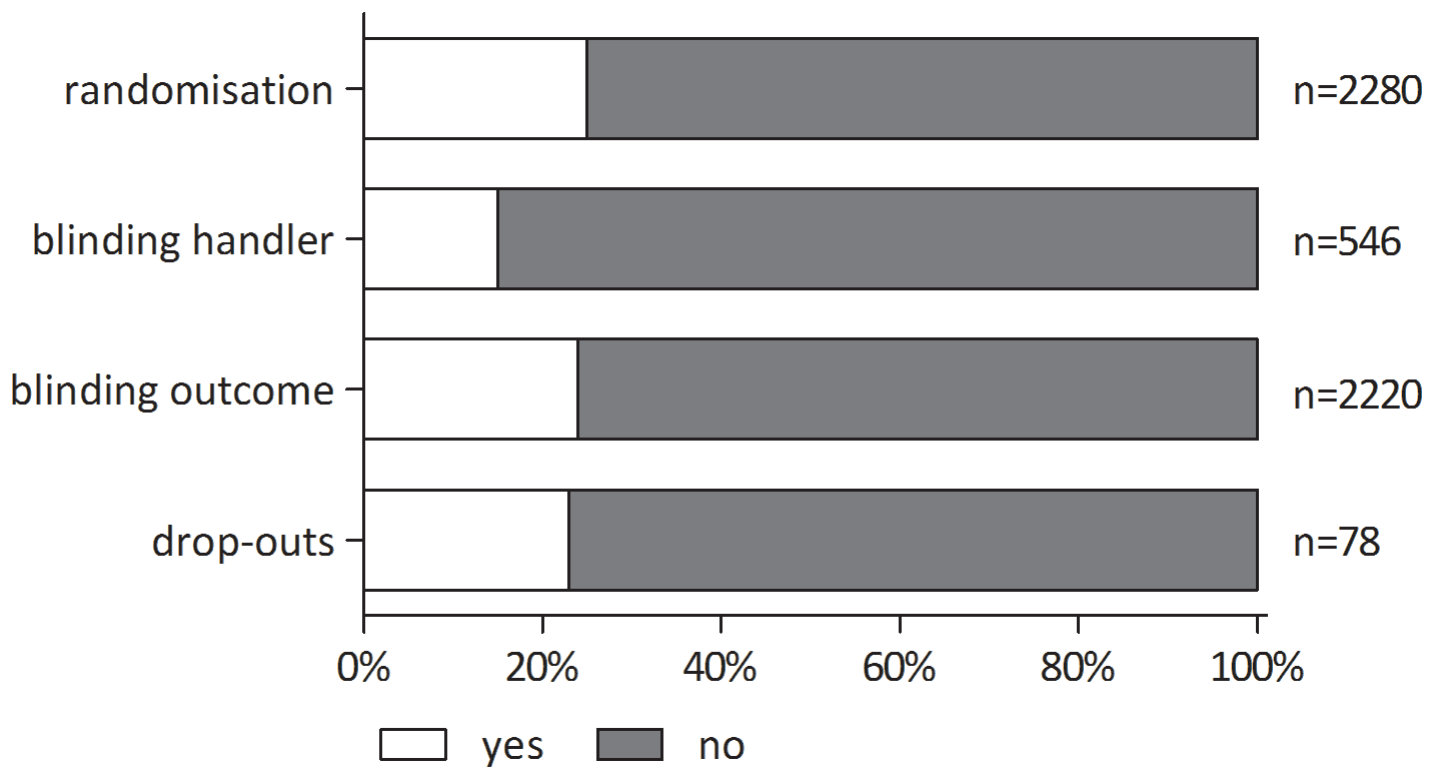
Percentage of primary animal studies assessed per validity item; yes or no.

Figure 4 provides an overview of risk of bias scores of the individual animal studies per item (randomisation, blinding of caretaker/investigator, blinding of outcome assessment and drop-outs). As not all reviews scored all four items we evaluated (see figure 3), the number of primary studies varies per item in figure 4. Of the 2280 included primary studies, 562 (24.6%) were randomised. Blinding of the investigator/caretaker was scored for 546 (23.9%) primary studies, of which 80 (14.6%) were actually blinded. Blinding of the outcome assessment was scored for 2220 (97.4%) primary studies, of which 530 (23.9%) were indeed blinded. Drop-outs were scored in only 78 (3.4%) primary studies, of which 18 (23.1%) really did reported drop-outs. One study assessed blinding, without specifying the type of blinding. Therefore, the data of this study were not included in our results (see Table S1 and S2 in File S2).

## Discussion

Our results show that the assessment of the methodological quality by systematic reviews of animal studies is quite poor. Half of the 91 evaluated SRs did not critically appraise the risk of bias in the included studies. Furthermore, the thirty-three reviews that did assess and report the risk of bias showed that the internal validity of most individual animal studies is poor as well. Therefore, there is a real risk that the outcomes of both, the individual studies and the subsequent SRs of these studies are biased.

Our findings that the methodological quality of SRs is poor are in line with findings by Peters et al., who identified a number of deficiencies in the conduct and reporting of SRs and meta-analyses of animal studies. Peters et al. suggest that initiatives to improve the conduct and reporting of primary animal studies and of SRs of animal studies should go hand-in-hand [2]. Poor internal validity of animal studies has previously been demonstrated by Kilkenny et al. Of the 271 publications of animal studies they surveyed, only 13% had been randomised and 14% had blinded the outcome assessment [9]. We found slightly higher percentages, namely 24.6% randomisation and 23.9% for blinding. These higher percentages may be explained by two factors. First, our study contains a relative high number of stroke studies. Over the last decades, researchers in the field of stroke have been actively working on recommendations and guidelines for preclinical research in order to improve effective translation [10]. Second, over the last years, general awareness of the need for better reporting of animal studies has been steadily increasing.

Although both the methodological quality of animal SRs and the internal validity of primary animal studies have been investigated before, they were studied separately by different research groups and more recent SRs of animal studies have not yet been taken into account. A major strength of our study is, therefore, that by updating and combining these evaluations in one study, we were able to gain more in-depth insight into the current state and level of available preclinical evidence.

Some potential limitations should also be discussed. First, we have restricted ourselves to one type of SR, namely SRs of animal-based drug-intervention studies, which might hamper the generalization of our results to other SRs of animal studies. Although we excluded SRs of animal studies that are not directly related to clinical research, we consider it likely that the latter type of SRs are of lower methodological quality, as the SR methodology and measures to safeguard internal validity may not be as well established as in fields closely related to clinical research. Therefore, our restriction might have caused an overestimation of the methodological quality of SRs and the internal validity of primary studies in general. Second, it cannot be ruled out that a small proportion of the SRs did not assess certain internal validity items, because the experimental design of the included individual studies did not allow a risk of bias assessment (e.g. due to a lack of (independent) control groups). Third, some individual studies may have been less subject to bias than the SRs estimated due to a lack of (adequate) *reporting* of the randomization and/or blinding methods they actually used. Fourth, we have not investigated whether the SRs assessed the adequacy of the *method* of randomisation or blinding. Inadequate randomization and blinding in animal studies can cause overestimation of the effect size [11], [12] and thus may falsely inform other preclinical research or clinical trials. In principle, this means that even randomised studies could be subject to bias, namely when the randomisation method was not adequate for the study design. Similarly, some SRs assessed blinding but did not specify the level or type of blinding. As long as the reporting of animal studies remains poor, however, these limitations are hardly avoidable.

Adequate internal validity of animal studies has been described as one of the key factors for improving the translation of results to human studies [1]. SRs can be a useful method to evaluate and analyse (the quality of) available evidence. As previously stated, SRs of animal studies could profit from the use of guidelines [2]. Currently, there is no standard procedure available for conducting SRs of animal studies [8], [13]. This could be one of the reasons why so many animal-based SRs did not assess any of the risk of bias items. Valuable lessons can be learned here from the guidelines used in clinical research, such as the CONSORT and PRISMA statements. Guidelines for planning, conducting and reporting primary animal studies are already available [14], [15]. Even though the ARRIVE guidelines are adopted by many journals, the effect on publication standards of animal studies is still very minimal. Therefore, effective implementation of endorsement of these guidelines requires more attention [16]. As does education on this matter. A good education strategy regarding both the internal validity of animal studies and the SR methodology can help raise awareness for the current state of potentially biased animal data. Authors, as well as reviewers and editors, need to be aware of the potential risk of this bias in animal studies and how it can adequately be reduced to eventually produce high-quality research with reliable results for human healthcare.

## Conclusions

To improve the translation of animal data to clinical practice, systematic reviews of animal studies are worthwhile, but the internal validity of the individual animal studies needs to be improved. Furthermore, risk of bias should be assessed by SRs of animal studies to provide insight into the reliability of available evidence.

## Supporting Information

File S1 **Search filters for Systematic reviews in PubMed and Embase.**
(DOC)

File S2 **This file contains Table S1 and Table S2.** Table S1, SR characteristics. Table S2, Internal validity included primary studies.
(DOC)

Checklist S1 **PRISMA Checklist.**
(DOC)
