# Supplementary material for: Systematic Reviews of Animal Studies; Missing Link in Translational Research?
Source: PLoS One. 2014 Mar 26;9(3):e89981. doi: 10.1371/journal.pone.0089981 (PMC3966727; doi:10.1371/journal.pone.0089981)
Supplement: File S1 — Search filters for Systematic reviews in PubMed and Embase. (DOC) [file pone.0089981.s001.doc]

**Systematic Review filters**

Pubmed:

(systematic review [ti] OR meta-analysis [pt] OR meta-analysis [ti] OR systematic literature review [ti] OR (systematic review [tiab] AND review [pt]) OR consensus development conference [pt] OR practice guideline [pt] OR cochrane database syst rev [ta] OR acp journal club [ta] OR health technol assess [ta] OR evid rep technol assess summ [ta]) OR ((evidence based[ti] OR evidence-based medicine [mh] OR best practice* [ti] OR evidence synthesis [tiab])AND (review [pt] OR diseases category[mh] OR behavior and behavior mechanisms [mh] OR therapeutics [mh] OR evaluation studies[pt] OR validation studies[pt] OR guideline [pt])) OR ((systematic [tw] OR systematically [tw] OR critical [tiab] OR (study selection [tw]) OR (predetermined [tw] OR inclusion [tw] AND criteri* [tw]) OR exclusion criteri* [tw] OR main outcome measures [tw] OR standard of care [tw] OR standards of care [tw]) AND (survey [tiab] OR surveys [tiab] OR overview* [tw] OR review [tiab] OR reviews [tiab] OR search* [tw] OR handsearch [tw] OR analysis [tiab] OR critique [tiab] OR appraisal [tw] OR (reduction [tw]AND (risk [mh] OR risk [tw]) AND (death OR recurrence))) AND (literature [tiab] OR articles [tiab] OR publications [tiab] OR publication [tiab] OR bibliography [tiab] OR bibliographies [tiab] OR published [tiab] OR unpublished [tw] OR citation [tw] OR citations [tw] OR database [tiab] OR internet [tiab] OR textbooks [tiab] OR references [tw] OR scales [tw] OR papers [tw] OR datasets [tw] OR trials [tiab] OR meta-analy* [tw] OR (clinical [tiab] AND studies [tiab]) OR treatment outcome [mh] OR treatment outcome [tw])) NOT (letter [pt] OR newspaper article [pt] OR comment [pt])

Embase:

(systematic review.ti. OR meta-analysis.pt. OR meta-analysis.ti. OR systematic literature review.ti. OR (systematic review.ti,ab. AND review.pt.) OR consensus development conference.pt. OR practice guideline.pt. OR cochrane database syst rev.ja. OR acp journal club.ja. OR health technol assess.ja. OR evid rep technol assess summ.ja.) OR ((evidence based.ti. OR evidence-based medicine/ OR best practice*.ti. OR evidence synthesis.ti,ab.) AND (review.pt. OR diseases category/ OR exp behavior and behavior mechanisms/ OR exp therapeutics/ OR evaluation studies.pt. OR validation studies.pt. OR guideline.pt.)) OR ((systematic.tw. OR systematically.tw. OR critical.ti,ab. OR (study selection.tw.) OR (predetermined.tw. OR inclusion.tw. AND criteri*.tw.) OR exclusion criteri*.tw. OR main outcome measures.tw. OR standard of care.tw. OR standards of care.tw.) AND (survey.ti,ab. OR surveys.ti,ab. OR overview*.tw. OR review.ti,ab. OR reviews.ti,ab. OR search*.tw. OR handsearch.tw. OR analysis.ti,ab. OR critique.ti,ab. OR appraisal.tw. OR (reduction.tw.AND (exp risk/ OR risk.tw.) AND (death OR recurrence))) AND (literature.ti,ab. OR articles.ti,ab. OR publications.ti,ab. OR publication.ti,ab. OR bibliography.ti,ab. OR bibliographies.ti,ab. OR published.ti,ab. OR unpublished.tw. OR citation.tw. OR citations.tw. OR database.ti,ab. OR internet.ti,ab. OR textbooks.ti,ab. OR references.tw. OR scales.tw. OR papers.tw. OR datasets.tw. OR trials.ti,ab. OR meta-analy*.tw. OR (clinical.ti,ab. AND studies.ti,ab.) OR exp treatment outcome/ OR treatment outcome.tw.)) NOT (letter.pt. OR newspaper article.pt. OR comment.pt.)
