# Supplementary material for: Systematic Reviews of Animal Studies; Missing Link in Translational Research?
Source: PLoS One. 2014 Mar 26;9(3):e89981. doi: 10.1371/journal.pone.0089981 (PMC3966727; doi:10.1371/journal.pone.0089981)
Supplement: File S2 — This file contains Table S1 and Table S2. Table S1, SR characteristics. Table S2, Internal validity included primary studies. (DOC) [file pone.0089981.s002.doc]

**Table 1 Characteristics SRs**

*Assessment of internal validity:*

1. Randomisation assessed
2. Blinding caretaker/investigator assessed
3. Blinding outcome assessment assessed
4. Dropouts assessed

*Use of internal validity:*

1. General comment
2. Used in meta-analysis (e.g. subgroup)
3. Exclusion criterion

*Additional information:*

1. Included in analyses of primary studies
2. Field of research

| **Reference** | **1** | **2** | **3** | **4** | **5** | **6** | **7** | **8** | **9** |
| --- | --- | --- | --- | --- | --- | --- | --- | --- | --- |
|  | * |  | * | * | * |  |  | * | Endocrinology |
|  | * |  | * |  | * | * |  | * | Neurology |
|  | * |  | * | * | * | * |  |  | Oncology |
|  |  |  |  |  |  |  |  |  | Endocrinology |
|  | * | * | * |  | * | * |  | * | Stroke |
|  | * | * | * | * | * | * |  | * | Stroke |
|  | * |  | * |  | * |  |  | * | Neurology |
|  |  |  |  |  |  |  |  |  | Cardiovascular Research |
|  | * |  | * | * | * |  |  | * | Orthopeadics |
|  | * | * | * |  | * | * |  | * | Stroke |
|  |  |  |  |  |  |  |  |  | Neurology |
|  | * |  |  |  |  |  | * |  | Pharmacotoxicology |
|  |  |  |  |  |  |  |  |  | Oncology |
|  |  |  |  |  |  |  |  |  | Infectious Diseases |
|  | * |  | * | * | * |  | * | * | Dentistry |
|  | * |  | * |  | * | * |  | * | Stroke |
|  |  |  |  |  |  |  |  |  | Infectious Diseases |
|  | * |  |  |  | * |  |  |  | Dentistry |
|  |  |  |  |  |  |  |  |  | Neurology |
|  |  |  |  |  |  |  |  |  | Pharmacotoxicology |
|  |  |  |  |  |  |  |  |  | Oncology |
|  | * |  |  |  |  |  |  |  | Neurology |
|  | * | * | * |  | * | * |  |  | Stroke |
|  | * |  | * |  | * | * |  | * | Neurology |
|  | * |  | * |  | * | * |  | * | Neurology |
|  | * |  | * |  | * | * |  | * | Stroke |
|  |  |  |  |  |  | * |  |  | Stroke |
|  | * |  |  |  | * |  |  |  | Orthopeadics |
|  | * |  | * |  |  |  |  |  | Stroke |
|  |  |  |  |  |  |  |  |  | Pharmacology |
|  |  |  |  |  |  |  |  |  | Endocrinology |
|  |  |  |  |  |  |  |  |  | Endocrinology |
|  |  |  |  |  |  |  |  |  | Pharmacotoxicology |
|  | * |  |  |  | * |  |  |  | Orthopeadics |
|  | * | * | * | * | * | * |  | * | Gastroenterology |
|  | * |  | * | * | * |  |  | * | Neurology |
|  | * |  |  |  | * |  |  | * | Infectious Diseases |
|  | * | * | * |  | * | * |  |  | Stroke |
|  |  |  |  |  |  |  |  |  | Dentistry |
|  | * | * | * |  | * |  |  | * | Neurology |
|  |  |  |  |  |  |  |  |  | Orthopeadics |
|  | * |  | * |  | * |  |  |  | Oncology |
|  |  |  |  |  |  |  |  |  | Oncology |
|  |  |  |  |  |  |  |  |  | Psychiatry |
|  |  |  |  |  |  |  |  |  | Neurology |
|  |  |  |  |  |  |  |  |  | Neurology |
|  | * | * | * |  | * | * |  | * | Stroke |
|  |  |  |  |  |  |  |  |  | Endocrinology |
|  |  |  |  |  |  |  |  |  | Endocrinology |
|  |  |  |  |  |  |  |  |  | Infectious Diseases |
|  | * | * | * |  | * | * |  | * | Cardiovascular Research |
|  | * |  | * |  | * |  |  | * | Stroke |
|  |  |  |  |  |  |  |  |  | Neurology |
|  | * | * | * |  | * |  |  | * | Stroke |
|  | * | * | * |  | * | * |  | * | Stroke |
|  | * | * | * |  | * | * |  | * | Stroke |
|  | * |  | * |  |  |  |  |  | Cardiovascular Research |
|  |  |  |  |  |  |  |  |  | Orthopeadics |
|  | * | * | * |  | * |  |  | * | Endocrinology |
|  |  |  |  |  |  |  |  |  | Orthopeadics |
|  | * |  |  |  |  |  | * |  | Gastroenterology |
|  | * | * | * |  | * |  |  |  | Stroke |
|  |  |  |  |  |  |  |  |  | Ophthalmology |
|  |  |  |  |  |  |  |  |  | Pharmacotoxicology |
|  |  |  |  |  |  |  |  |  | Infectious Diseases |
|  |  |  |  |  | * |  |  |  | Cardiovascular Research |
|  |  |  |  |  |  |  |  |  | Surgery |
|  |  |  |  |  |  |  |  |  | Endocrinology |
|  | * |  | * |  | * | * |  | * | Neurology |
|  |  |  |  |  |  |  |  |  | Gastroenterology |
|  |  |  |  |  |  |  |  |  | Endocrinology |
|  | * | * | * |  | * | * |  | * | Stroke |
|  | * | * | * |  | * | * |  | * | Stroke |
|  | * |  |  |  | * |  |  | * | Endocrinology |
|  |  |  |  |  |  |  |  |  | Neurology |
|  |  |  |  |  |  |  |  |  | Neurology |
|  |  |  |  |  |  |  |  |  | Neurology |
|  |  |  |  |  |  |  |  |  | Pulmonology |
|  | * | * | * |  | * |  |  | * | Cardiovascular Research |
|  |  |  |  |  |  |  |  |  | Pharmacotoxicology |
|  | * |  | * |  | * | * |  | * | Neurology |
|  |  |  |  |  |  |  |  |  | Otolaryngology |
|  |  |  |  |  |  |  |  |  | Nephrology |
|  |  |  |  |  | * |  |  |  | Pulmonology |
| **[[1]](#footnote-2)** | * | ? | ? |  |  |  |  | * | Cardiovascular Research |
|  | * | * | * |  | * | * |  | * | Stroke |
|  | * |  |  |  | * | * |  |  | Stroke |
|  | * |  | * |  | * | * |  |  | Stroke |
|  |  |  |  |  |  |  |  |  | Surgery |
|  | * | * | * | * | * |  |  | * | Endocrinology |
|  | * | * | * |  | * | * |  | * | Neurology |
| **n=** | **48** | **38** | **20** | **8** | **44[[2]](#footnote-3)** | **25** | **3** | **33** |  |

**Table 2 Primary animal studies**

1. Total number of animals included
2. Number of animals randomized
3. Number of animals blinded caretaker/investigator
4. Number of animals blinded outcome assessment
5. Number of animals drop-outs

? = number unclear

- = not scored

| **Reference** | **1** | **2** | **3** | **4** | **5** |
| --- | --- | --- | --- | --- | --- |
|  | 11 | 5 | 0 | - | 1 |
|  | 62 | 45 | 33 | - | - |
|  | 17 | 1 | 5 | 2 | - |
|  | 18 | 9 | 12 | 8 | 9 |
|  | 85 | 21 | 21 | - | - |
|  | 6 | 3 | 2 | - | 0 |
|  | 14 | 6 | 7 | 5 | - |
|  | 2 | 2 | 2 | - | 2 |
|  | 19 | 16 | 16 | - | - |
|  | 12 | 2 | 5 | - | - |
|  | 18 | 12 | 7 | - | - |
|  | 27 | 2 | 1 | - | - |
|  | 13 | 10 | 7 | 2 | 4 |
|  | 17 | 13 | 1 | - | 1 |
|  | 2 | 0 | - | - | - |
|  | 14 | 7 | 4 | 0 | - |
|  | 117 | 54 | 57 | 22 | - |
|  | 20 | 15 | 2 | 1 | - |
|  | 9 | 4 | 4 | - | - |
|  | 25 | 6 | 7 | 0 | - |
|  | 13 | 4 | 4 | 2 | - |
|  | 29 | 6 | 2 | 1 | - |
|  | 4 | 0 | 0 | 0 | - |
|  | 254 | 40 | 38 | - | - |
|  | 105 | 46 | 28 | 16 | - |
|  | 18 | 12 | 13 | 1 | - |
|  | 13 | 12 | - | - | - |
|  | 52 | 38 | 22 | 5 | - |
|  | 1152 | 108 | 184 | - | - |
| **[[3]](#footnote-4)** | 45 | 12 | ? | ? | - |
|  | 6 | 2 | 1 | 0 | - |
|  | 11 | 11 | 0 | 0 | 1 |
|  | 70 | 38 | 45 | 15 | - |
|  |  |  |  |  |  |

**Systematic Review References**

1. Ainge, H., et al., *A systematic review on animal models of maternal high fat feeding and offspring glycaemic control.* Int J Obes (Lond), 2011. **35**(3): p. 325-35.

2. Akhtar, A.Z., J.J. Pippin, and C.B. Sandusky, *Animal studies in spinal cord injury: A systematic review of methylprednisolone.* ATLA Alternatives to Laboratory Animals, 2009. **37**(1): p. 43-62.

3. Amarasingh, S., M.R. Macleod, and I.R. Whittle, *What is the translational efficacy of chemotherapeutic drug research in neuro-oncology? A systematic review and meta-analysis of the efficacy of BCNU and CCNU in animal models of glioma.* J Neurooncol, 2009. **91**(2): p. 117-25.

4. Auvinen, H.E., et al., *Effects of high fat diet on the Basal activity of the hypothalamus-pituitary-adrenal axis in mice: a systematic review.* Horm Metab Res, 2011. **43**(13): p. 899-906.

5. Banwell, V., E.S. Sena, and M.R. Macleod, *Systematic review and stratified meta-analysis of the efficacy of interleukin-1 receptor antagonist in animal models of stroke.* J Stroke Cerebrovasc Dis, 2009. **18**(4): p. 269-76.

6. Baryan, H.K., et al., *Systematic review and meta-analysis of the efficacy of statins in experimental stroke.* Int J Stroke, 2012. **7**(2): p. 150-6.

7. Benatar, M., *Lost in translation: treatment trials in the SOD1 mouse and in human ALS.* Neurobiol Dis, 2007. **26**(1): p. 1-13.

8. Berthelsen, L.O., A.T. Kristensen, and M. Tranholm, *Animal models of DIC and their relevance to human DIC: A systematic review.* Thrombosis research, 2011. **128 (2)**: p. 103-116.

9. Borhanuddin, B., N.F. Mohd Fozi, and I. Naina Mohamed, *Vitamin e and the healing of bone fracture: the current state of evidence.* Evid Based Complement Alternat Med, 2012. **2012**: p. 684510.

10. Bustamante, A., et al., *Citicoline in pre-clinical animal models of stroke: a meta-analysis shows the optimal neuroprotective profile and the missing steps for jumping into a stroke clinical trial.* J Neurochem, 2012. **123**(2): p. 217-25.

11. Cadotte, D.W. and M.G. Fehlings, *Spinal cord injury: a systematic review of current treatment options.* Clin Orthop Relat Res, 2011. **469**(3): p. 732-41.

12. Cave, G. and M. Harvey, *Intravenous lipid emulsion as antidote beyond local anesthetic toxicity: a systematic review.* Acad Emerg Med, 2009. **16**(9): p. 815-24.

13. Corpet, D.E. and F. Pierre, *How good are rodent models of carcinogenesis in predicting efficacy in humans? A systematic review and meta-analysis of colon chemoprevention in rats, mice and men.* Eur J Cancer, 2005. **41**(13): p. 1911-22.

14. Dare, A.J., et al., *A systematic review of experimental treatments for mitochondrial dysfunction in sepsis and multiple organ dysfunction syndrome.* Free Radic Biol Med, 2009. **47**(11): p. 1517-25.

15. Dhingra, K. and K.L. Vandana, *Prophylactic vaccination against periodontal disease: a systematic review of preclinical studies.* J Periodontol, 2010. **81**(11): p. 1529-46.

16. England, T.J., C.L. Gibson, and P.M.W. Bath, *Granulocyte-colony stimulating factor in experimental stroke and its effects on infarct size and functional outcome: A systematic review.* Brain Research Reviews, 2009. **62**(1): p. 71-82.

17. Eyers, S., et al., *The effect on mortality of antipyretics in the treatment of influenza infection: systematic review and meta-analysis.* Journal of the Royal Society of Medicine, 2010. **103**(10): p. 403-411.

18. Faggion, C.M., Jr., et al., *Comparison of the effects of treatment of peri-implant infection in animal and human studies: systematic review and meta-analysis.* Clin Oral Implants Res, 2010. **21**(2): p. 137-47.

19. Farooqi, N., B. Gran, and C. Constantinescu, *Are current disease-modifying therapeutics in multiple sclerosis justified on the basis of studies in experimental autoimmune encephalomyelitis?* Journal of Neurochemistry, 2010. **115**(4): p. 829-844.

20. Fond, G., et al., *Antipsychotic drugs: pro-cancer or anti-cancer? A systematic review.* Med Hypotheses, 2012. **79**(1): p. 38-42.

21. Fritz, H., et al., *Vitamin A and retinoid derivatives for lung cancer: a systematic review and meta analysis.* PLoS ONE, 2011. **6**(6): p. e21107.

22. Frogley, C., et al., *A systematic review of the evidence of clozapine's anti-aggressive effects.* Int J Neuropsychopharmacol, 2012. **15**(9): p. 1351-71.

23. Garcia-Bonilla, L., et al., *Evidence for the efficacy of statins in animal stroke models: a meta-analysis.* J Neurochem, 2012.

24. Gibson, C. and S. Murphy, *Benefits of histone deacetylase inhibitors for acute brain injury: A systematic review of animal studies.* Journal of Neurochemistry, 2010. **115**(4): p. 806-813.

25. Gibson, C.L., et al., *Progesterone for the treatment of experimental brain injury; a systematic review.* Brain, 2008. **131**(2): p. 318-328.

26. Gibson, C.L., et al., *Estrogens and experimental ischemic stroke: a systematic review.* J Cereb Blood Flow Metab, 2006. **26**(9): p. 1103-13.

27. Gibson, C.L., A.N. Murphy, and S.P. Murphy, *Stroke outcome in the ketogenic state--a systematic review of the animal data.* J Neurochem, 2012. **123 Suppl 2**: p. 52-7.

28. Gielkens, P.F., et al., *Is there evidence that barrier membranes prevent bone resorption in autologous bone grafts during the healing period? A systematic review.* Int J Oral Maxillofac Implants, 2007. **22**(3): p. 390-8.

29. Harston, G., et al., *The contribution of L-arginine to the neurotoxicity of recombinant tissue plasminogen activator following cerebral ischemia: A review of rtPA neurotoxicity.* Journal of Cerebral Blood Flow and Metabolism, 2010. **30**(11): p. 1804-1816.

30. Hasani-Ranjbar, S., B. Larijani, and M. Abdollah, *A systematic review of iranian medicinal plants useful in diabetes mellitus.* Archives of Medical Science, 2008. **4**(3): p. 285-292.

31. Hasani-Ranjbar, S., B. Larijani, and M. Abdollahi, *A systematic review of the potential herbal sources of future drugs effective in oxidant-related diseases.* Inflamm Allergy Drug Targets, 2009. **8**(1): p. 2-10.

32. Hasani-Ranjbar, S., et al., *A systematic review of the efficacy and safety of herbal medicines used in the treatment of obesity.* World J Gastroenterol, 2009. **15**(25): p. 3073-85.

33. Heard, K., N.R. Cleveland, and S. Krier, *Benzodiazepines and antipsychotic medications for treatment of acute cocaine toxicity in animal models--a systematic review and meta-analysis.* Hum Exp Toxicol, 2011. **30**(11): p. 1849-54.

34. Hirsch, B.P., et al., *The effect of therapies for osteoporosis on spine fusion: a systematic review.* Spine J, 2012.

35. Hooijmans, C.R., et al., *The effects of probiotic supplementation on experimental acute pancreatitis: a systematic review and meta-analysis.* PLoS One, 2012. **7**(11): p. e48811.

36. Hooijmans, C.R., et al., *The effects of long-term omega-3 fatty acid supplementation on cognition and Alzheimer's pathology in animal models of Alzheimer's disease: a systematic review and meta-analysis.* J Alzheimers Dis, 2012. **28**(1): p. 191-209.

37. Jefferies, S., et al., *Systematic review and meta-analysis of the effects of antipyretic medications on mortality in Streptococcus pneumoniae infections.* Postgrad Med J, 2012. **88**(1035): p. 21-7.

38. Jerndal, M., et al., *A systematic review and meta-analysis of erythropoietin in experimental stroke.* Journal of Cerebral Blood Flow and Metabolism, 2010. **30**(5): p. 961-968.

39. Jung, R.E., D.S. Thoma, and C.H. Hammerle, *Assessment of the potential of growth factors for localized alveolar ridge augmentation: a systematic review.* J Clin Periodontol, 2008. **35**(8 Suppl): p. 255-81.

40. Ker, K., P. Perel, and K. Blackhall, *Beta-2 receptor antagonists for traumatic brain injury: A systematic review of controlled trials in animal models.* CNS Neuroscience and Therapeutics, 2009. **15**(1): p. 52-64.

41. Khojasteh, A., et al., *Effects of different growth factors and carriers on bone regeneration: a systematic review.* Oral Surg Oral Med Oral Pathol Oral Radiol, 2012.

42. Kienle, G.S., et al., *Viscum album L. extracts in breast and gynaecological cancers: a systematic review of clinical and preclinical research.* J Exp Clin Cancer Res, 2009. **28**: p. 79.

43. Kienle, G.S., R. Grugel, and H. Kiene, *Safety of higher dosages of Viscum album L. in animals and humans--systematic review of immune changes and safety parameters.* BMC Complement Altern Med, 2011. **11**: p. 72.

44. Kontis, D. and E. Theochari, *Dopamine in anorexia nervosa: a systematic review.* Behav Pharmacol, 2012. **23**(5-6): p. 496-515.

45. Kwon, B.K., et al., *A systematic review of non-invasive pharmacologic neuroprotective treatments for acute spinal cord injury.* J Neurotrauma, 2011. **28**(8): p. 1545-88.

46. Kwon, B.K., et al., *A systematic review of directly applied biologic therapies for acute spinal cord injury.* J Neurotrauma, 2011. **28**(8): p. 1589-610.

47. Lees, J.S., et al., *Stem cell-based therapy for experimental stroke: a systematic review and meta-analysis.* Int J Stroke, 2012. **7**(7): p. 582-8.

48. Lerchbaum, E. and B. Obermayer-Pietsch, *Vitamin D and fertility: a systematic review.* Eur J Endocrinol, 2012. **166**(5): p. 765-78.

49. Lerchbaum, E. and B. Obermayer-Pietsch, *Mechanisms in endocrinology - Vitamin D and fertility: A systematic review.* European Journal of Endocrinology, 2012. **166 (5)**: p. 765-778.

50. Li, Y., et al., *The effect of heparin administration in animal models of sepsis: a prospective study in Escherichia coli-challenged mice and a systematic review and metaregression analysis of published studies.* Crit Care Med, 2011. **39**(5): p. 1104-12.

51. Lim, W.Y., C.M. Messow, and C. Berry, *Cyclosporin variably and inconsistently reduces infarct size in experimental models of reperfused myocardial infarction: a systematic review and meta-analysis.* Br J Pharmacol, 2012. **165**(7): p. 2034-43.

52. Liu, J., et al., *Systematic Review and Meta-Analysis of the Efficacy of Sphingosine-1-Phosphate (S1P) Receptor Agonist FTY720 (Fingolimod) in Animal Models of Stroke.* Int J Neurosci, 2012.

53. Loef, M., G.N. Schrauzer, and H. Walach, *Selenium and Alzheimer's disease: a systematic review.* J Alzheimers Dis, 2011. **26**(1): p. 81-104.

54. MacDougall, N.J. and K.W. Muir, *Hyperglycaemia and infarct size in animal models of middle cerebral artery occlusion: systematic review and meta-analysis.* J Cereb Blood Flow Metab, 2011. **31**(3): p. 807-18.

55. Macleod, M.R., et al., *Systematic review and metaanalysis of the efficacy of FK506 in experimental stroke.* Journal of Cerebral Blood Flow and Metabolism, 2005. **25**(6): p. 713-721.

56. Macleod, M.R., et al., *Systematic review and meta-analysis of the efficacy of melatonin in experimental stroke.* Journal of Pineal Research, 2005. **38**(1): p. 35-41.

57. Matthan, N.R., et al., *A systematic review and meta-analysis of the impact of omega-3 fatty acids on selected arrhythmia outcomes in animal models.* Metabolism, 2005. **54**(12): p. 1557-65.

58. Mehrkens, A., et al., *Tissue engineering approaches to degenerative disc disease--a meta-analysis of controlled animal trials.* Osteoarthritis Cartilage, 2012. **20**(11): p. 1316-25.

59. Muhlhausler, B.S., R.A. Gibson, and M. Makrides, *The effect of maternal omega-3 long-chain polyunsaturated fatty acid (n-3 LCPUFA) supplementation during pregnancy and/or lactation on body fat mass in the offspring: a systematic review of animal studies.* Prostaglandins Leukot Essent Fatty Acids, 2011. **85**(2): p. 83-8.

60. Naina Mohamed, I., et al., *Vitamin e and bone structural changes: an evidence-based review.* Evid Based Complement Alternat Med, 2012. **2012**: p. 250584.

61. Nicholson, I., A.M. Dalzell, and W. El-Matary, *Vitamin D as a therapy for colitis: a systematic review.* J Crohns Colitis, 2012. **6**(4): p. 405-11.

62. O'Collins, V.E., et al., *Preclinical drug evaluation for combination therapy in acute stroke using systematic review, meta-analysis, and subsequent experimental testing.* J Cereb Blood Flow Metab, 2011. **31**(3): p. 962-75.

63. Papathanassiou, M., et al., *Vascular Endothelial Growth Factor Inhibitors for Treatment of Corneal Neovascularization: A Meta-Analysis.* Cornea, 2012.

64. Percie du Sert, N., et al., *Cisplatin-induced emesis: systematic review and meta-analysis of the ferret model and the effects of 5-HT receptor antagonists.* Cancer Chemother Pharmacol, 2011. **67**(3): p. 667-86.

65. Perlroth, J., et al., *Adjunctive use of rifampin for the treatment of Staphylococcus aureus infections: A systematic review of the literature.* Archives of Internal Medicine, 2008. **168**(8): p. 805-819.

66. Pilcher, J., et al., *The effect of hyperoxia following cardiac arrest - A systematic review and meta-analysis of animal trials.* Resuscitation, 2012. **83**(4): p. 417-22.

67. Pommergaard, H.C., M.P. Achiam, and J. Rosenberg, *External coating of colonic anastomoses: a systematic review.* Int J Colorectal Dis, 2012. **27**(10): p. 1247-58.

68. Ranasinghe, P., et al., *Efficacy and safety of 'true' cinnamon (Cinnamomum zeylanicum) as a pharmaceutical agent in diabetes: a systematic review and meta-analysis.* Diabet Med, 2012. **29**(12): p. 1480-92.

69. Rooke, E.D., et al., *Dopamine agonists in animal models of Parkinson's disease: a systematic review and meta-analysis.* Parkinsonism Relat Disord, 2011. **17**(5): p. 313-20.

70. Rossignol, D.A., *Hyperbaric oxygen treatment for inflammatory bowel disease: a systematic review and analysis.* Med Gas Res, 2012. **2**(1): p. 6.

71. Rumore, M.M. and K.S. Kim, *Potential role of salicylates in type 2 diabetes.* Ann Pharmacother, 2010. **44**(7-8): p. 1207-21.

72. Sena, E., et al., *Systematic review and meta-analysis of the efficacy of tirilazad in experimental stroke.* Stroke, 2007. **38**(2): p. 388-394.

73. Sena, E.S., et al., *Factors affecting the apparent efficacy and safety of tissue plasminogen activator in thrombotic occlusion models of stroke: systematic review and meta-analysis.* J Cereb Blood Flow Metab, 2010. **30**(12): p. 1905-13.

74. Sun, G., et al., *Adoptive infusion of tolerogenic dendritic cells prolongs the survival of pancreatic islet allografts: a systematic review of 13 mouse and rat studies.* PLoS One, 2012. **7**(12): p. e52096.

75. Tetzlaff, W., et al., *A systematic review of cellular transplantation therapies for spinal cord injury.* J Neurotrauma, 2011. **28**(8): p. 1611-82.

76. Trevitt, C.R. and J. Collinge, *A systematic review of prion therapeutics in experimental models.* Brain, 2006. **129**(9): p. 2241-2265.

77. Tsang, H. and T. Ho, *A systematic review on the anxiolytic effects of aromatherapy on rodents under experimentally induced anxiety models.* Reviews in the neurosciences, 2010. **21**(2): p. 141-152.

78. Tuinman, P.R., et al., *Nebulized anticoagulants for acute lung injury - a systematic review of preclinical and clinical investigations.* Crit Care, 2012. **16**(2): p. R70.

79. van der Spoel, T.I., et al., *Human relevance of pre-clinical studies in stem cell therapy: systematic review and meta-analysis of large animal models of ischaemic heart disease.* Cardiovasc Res, 2011. **91**(4): p. 649-58.

80. Vang, O., et al., *What is new for an old molecule? Systematic review and recommendations on the use of resveratrol.* PLoS ONE, 2011. **6 (6)**: p. e19881.

81. Vesterinen, H.M., et al., *Improving the translational hit of experimental treatments in multiple sclerosis.* Mult Scler, 2010. **16**(9): p. 1044-55.

82. Waissbluth, S., J. Pitaro, and S.J. Daniel, *Gene therapy for cisplatin-induced ototoxicity: a systematic review of in vitro and experimental animal studies.* Otol Neurotol, 2012. **33**(3): p. 302-10.

83. Wang, Y., et al., *Systematic Review and Meta-analysis of MSC Therapy for Impaired Renal Function in Small Animal Models.* Nephrology (Carlton), 2012.

84. Warner, D. and S.E. Brietzke, *Mitomycin C and airway surgery: How well does it work?* Otolaryngology Head and Neck Surgery, 2008. **138**(6): p. 700-709.

85. Weigl, M., et al., *A systematic review of currently available pharmacological neuroprotective agents as a sole intervention before anticipated or induced cardiac arrest.* Resuscitation, 2005. **65**(1): p. 21-39.

86. Wheble, P.C.R., E.S. Sena, and M.R. Macleod, *A systematic review and meta-analysis of the efficacy of piracetam and piracetam-like compounds in experimental stroke.* Cerebrovascular Diseases, 2008. **25**(1-2): p. 5-11.

87. White, A. and A. Murphy, *Administration of thiazolidinediones for neuroprotection in ischemic stroke: A pre-clinical systematic review.* Journal of Neurochemistry, 2010. **115**(4): p. 845-853.

88. Willmot, M., et al., *Nitric oxide synthase inhibitors in experimental ischemic stroke and their effects on infarct size and cerebral blood flow: A systematic review.* Free Radical Biology and Medicine, 2005. **39**(3): p. 412-425.

89. Yiannakopoulou, E., et al., *Pharmacological modulation of oxidative stress response in minimally invasive surgery: systematic review.* Surg Laparosc Endosc Percutan Tech, 2012. **22**(3): p. 200-4.

90. Zhang, J., et al., *Systematic review of the renal protective effect of Astragalus membranaceus (root) on diabetic nephropathy in animal models.* Journal of Ethnopharmacology, 2009. **126**(2): p. 189-196.

91. Zoerle, T., et al., *Pharmacologic reduction of angiographic vasospasm in experimental subarachnoid hemorrhage: systematic review and meta-analysis.* J Cereb Blood Flow Metab, 2012. **32**(9): p. 1645-58.

1. Level of blinding was unclear [↑](#footnote-ref-2)
2. In two reviews a comment was made about internal validity without a risk of bias assessment [↑](#footnote-ref-3)
3. Level of blinding unclear (not specified). Of the 45 primary studies: 15 were blinded (33.3%), 14 not blinded (31.1%) and in 16 studies blinding was not mentioned (35.6%). [↑](#footnote-ref-4)
